# X66, a novel N-terminal heat shock protein 90 inhibitor, exerts antitumor effects without induction of heat shock response

**DOI:** 10.18632/oncotarget.8818

**Published:** 2016-04-18

**Authors:** Zhixin Zhao, Jianming Zhu, Haitian Quan, Guimin Wang, Bo Li, Weiliang Zhu, Chengying Xie, Liguang Lou

**Affiliations:** ^1^ Shanghai Institute of Materia Medica, Chinese Academy of Sciences, Shanghai, 201203, China

**Keywords:** X66, HSP90, HSF-1, heat shock response, celastrol

## Abstract

Heat shock protein 90 (HSP90) is essential for cancer cells to assist the function of various oncoproteins, and it has been recognized as a promising target in cancer therapy. Although the HSP90 inhibitors in clinical trials have shown encouraging clinical efficacy, these agents induce heat shock response (HSR), which undermines their therapeutic effects. In this report, we detailed the pharmacologic properties of 4-(2-((1H-indol-3-yl)methylene)hydrazinyl)-N-(4-bromophenyl)-6-(3,5- dimethyl-1H -pyrazol-1-yl)-1,3,5-triazin-2-amine (X66), a novel and potent HSP90 inhibitor. X66 binds to the N-terminal domain in a different manner from the classic HSP90 inhibitors. Cellular study showed that X66 depleted HSP90 client proteins, resulted in cell cycle arrest and apoptosis, and inhibition of proliferation in cancer cell lines. X66 did not activate heat shock factor-1 (HSF-1) or stimulate transcription of HSPs. Moreover, the combination of X66 with HSP90 and proteasome inhibitors yielded synergistic cytotoxicity which was involved in X66-mediated abrogation of HSR through inhibition of HSF-1 activity. The intraperitoneal administration of X66 alone depleted client protein and inhibited tumor growth, and led to enhanced activity when combined with celastrol as compared to either agent alone in BT-474 xenograft model. Collectively, the HSP90 inhibitory action and the potent antitumor activity, with the anti-HSR action, promise X66 a novel HSP90-targeted agent, which merits further research and development.

## INTRODUCTION

The molecular chaperone heat shock protein 90 (HSP90) is essential for the viability of eukaryotic cells. HSP90, in complex with other cochaperone proteins [1, 2], directs the maturation and stabilization of an array of proteins referred to as client proteins [3], via its ATPase activity [4]. A number of these proteins are oncogenic proteins, including tyrosine-kinase receptors, signal transduction proteins and transcription factors, which are crucial for the development and promotion of cancer. Since cancer cells require HSP90 to maintain its homeostasis by stabilizing the mutated or overexpressed oncoproteins, HSP90 has become a promising target for cancer therapy.

The first HSP90 inhibitor geldanamycin (GM) [5], and the inhibitors in clinical trials, including GM analogues, resocinol derivatives, purine analogues and other compounds, bind to an ATP pocket in the N-terminal domain [6]. The binding inhibits HSP90 ATPase activity and results in the degradation of oncogenic client proteins through ubiquitin-mediated proteasomal degradation [7, 8]. The degradation leads to tumor cell growth arrest and activation of apoptosis [9]. The compounds targeting the ATP pocket in HSP90 N-terminal domain are referred to as classic HSP90 inhibitors.

Although some of classic HSP90 inhibitors have obtained positive results in clinical trials [10, 11], they will induce heat shock response (HSR), which is activated mainly by heat shock factor-1 (HSF-1), a transcription factor repressed by HSP90 complex under normal conditions [12, 13]. After the treatment with HSP90 inhibitor, the interaction of monomeric HSF-1 with HSP90 complex is disrupted. This results in the up-regulation of a wide range of heat shock proteins (HSPs), including HSP72 and HSP27 [14, 15], both of which are anti-apoptotic and tumorigenic [16, 17]. Consequently, targeting HSR is a promising strategy for enhancing therapeutic efficacy on HSP90 inhibition. Evidence has been accumulated that inhibition of HSP72 or HSP27 increases cellular sensitivity to HSP90 inhibitor [18–20]. In addition, studies of HSF-1 show the positive correlation between HSF-1 and tumor malignancy [21], and silencing HSF-1 increases antitumor activity of HSP90 inhibitor [22].

In this study, a triazine derivative, 4-(2-((1H-indol-3-yl)methylene) hydrazinyl)-N- (4-bromophenyl)-6-(3,5-dimethyl-1H-pyrazol-1-yl)-1,3,5-triazin-2-amine (X66)stood out as a novel N-terminal inhibitor with a unique HSP90 binding site which was distinct from all those known HSP90 inhibitors. We aimed to determine the anti-tumor activities of X66 both *in vitro* and *in vivo*, and figure out the related possible mechanisms of action. The results indicated that X66 depleted client proteins, caused cell-cycle arrest and apoptosis. More importantly, X66 did not induce HSR alone, and abrogated other chemotherapeutic compounds-induced HSR and potentiated the antitumor activity. These findings help to elucidate the precise mechanisms involved in the antitumor activities of X66 and enable the rational design of novel HSP90–targeted drugs.

## RESULTS

### X66 is a novel N-terminal HSP90 inhibitor

#### X66 is a potent and novel HSP90 inhibitor

**Figure 1:**
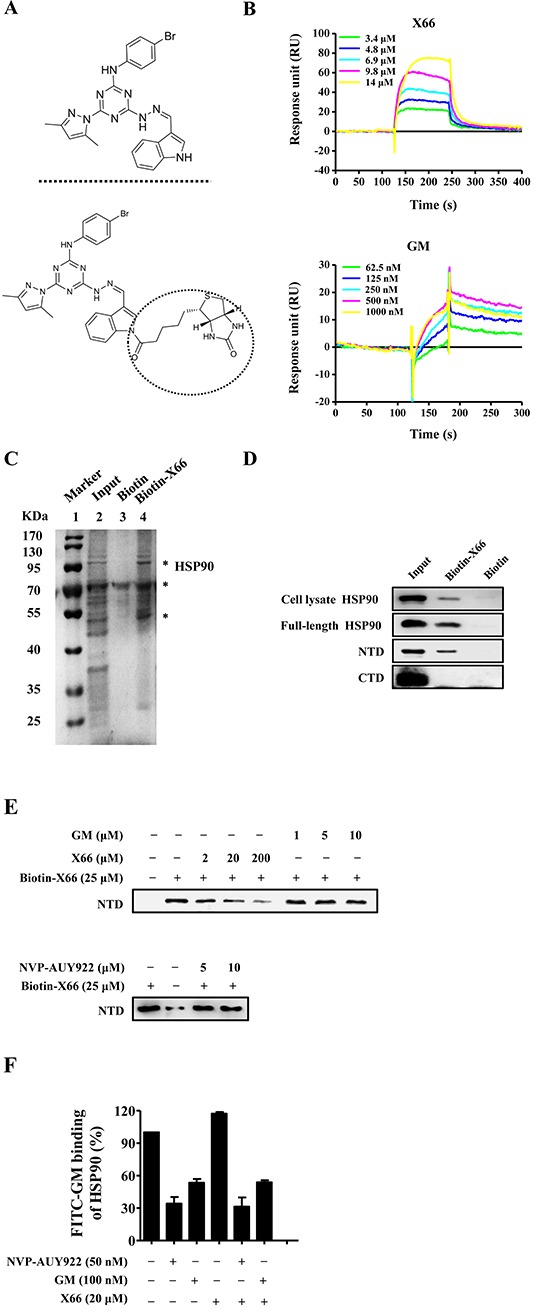
X66 is a novel N-terminal inhibitor of HSP90 **A.** Chemical structures of X66 (upper panel) and Biotin-X66 (lower panel). **B.** SPR analysis of X66 binding to full-length HSP90α. Sensorgrams obtained by injection of X66 and GM at the indicated concentrations on immobilized HSP90α. **C.** Bands isolated in the pull-down assay using SK-BR-3 cell lysate. Lane1: MW marker; lane 2: cell lysate input; lane 3: negative control (Biotin); Lane 4: Biotin-X66. Major proteins are indicated by asterisks. The first one is HSP90 followed the order from top to bottom. **D.** Cell lysates of SK-BR-3 cells, full-length HSP90α, fragment of N-terminal domain (NTD) and fragment of C-terminal domain (CTD) were tested for binding to Biotin-X66. The assay was carried out as described under “Materials and Methods”. HSP90 in cell lysates and full-length HSP90α were detected by HSP90 antibody, and NTD and CTD were detected by His-tag antibody. **E.** X66, GM and NVP-AUY922 in increasing concentrations were used to compete with Biotin-X66 on binding to NTD. **F.** FP assay was carried out as described under “Materials and Methods”. The HSP90α was incubated with GM, NVP-AUY922 or their combination with X66 at the indicated concentrations. n=3; Error bars ± SEM.

We used the surface plasmon resonance (SPR) assay to measure the direct interaction of HSP90 with its inhibitors, and identified X66 as a novel HSP90 inhibitor (Figure 1A). As shown in Figure 1B, both X66 and GM bound to the recombinant human full-length HSP90α immobilized on sensor chip in a concentration-dependent manner, yielding dissociation constants (K_D_) of 5.3 μM and 0.1 μM, respectively.

Next, the pull-down gel showed that Biotin-X66 was able to isolate HSP90 in SK-BR-3 cell lysates. The major proteins bound to Biotin-X66 were visualized with Coomassie blue as bands at approximately 95 KDa, 70 KDa and 55 KDa, respectively (Figure 1C). The protein appeared at 95 KDa had been identified as HSP90 by Western blot (Figure 1D).

#### X66 binds to a different N-terminal domain of HSP90 from that of classic HSP90 inhibitors

HSP90 has three structural domains: the N-terminal domain contains an ATP pocket and has ATPase activity, the middle domain provides binding sites for cochaperones and client proteins, and the C-terminal domain is responsible for HSP90 being a dimer and has a second ATP pocket [23]. The majority of HSP90 inhibitors bind to sites of the N-terminal domain or C-terminal domain. The *in vitro* binding assay showed that Biotin-X66 was able to isolate the recombinant human full-length protein and its N-terminal fragment, but not the C-terminal fragment (Figure 1D). The amount of HSP90 N-terminal fragment was directly proportional to the amount of Biotin-X66 used (data not shown), and diminished by preincubation with excess soluble X66 (Figure 1E). Unexpectedly, GM and the more potent HSP90 inhibitor NVP-AUY922 were unable to compete against Biotin-X66 for binding to the N-terminal fragment after preincubation with the protein solution. This result was further confirmed by the competitive binding fluorescence polarization (FP) assay. As shown in Figure 1F, X66 failed to block the interaction of FITC-GM, GM or NVP-AUY922 with the recombinant HSP90. Thus, these results suggest that X66 binds to the N-terminal domain of HSP90 in a manner that is different from classic HSP90 inhibitors.

### X66 inhibits tumor cell proliferations and induces cell cycle arrest and apoptosis

**Figure 2:**
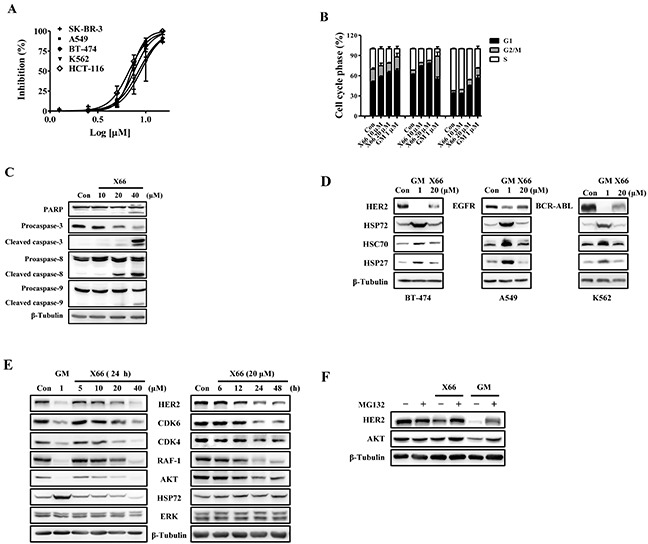
X66 inhibits proliferation of tumor cell lines and causes HSP90 client proteins degradation in vitro **A.** Anti-proliferative effects of X66 against SK-BR-3, BT-474, A549, HCT-116 and K562 cells. n=3; Error bars ± SEM. **B.** Representative cell cycle phase histograms of SK-BR-3, A549 and K562 cells following treatment with X66 or GM for 24 h, respectively. n=3; Error bars ± SEM. **C.** Levels of PARP, Procaspase-3, Caspase-8 and Caspase-9 were determined by Western blot in SK-BR-3 cells following 48-h exposure to increasing concentrations of X66. β-Tubulin was included as loading control in all experiments. **D.** Western blot analysis of BT-474, A549 or K562 cells following 24-h exposure to 20 μM X66 or 1 μM GM. **E.** Western blot analysis of SK-BR-3 following 24-h exposure to increasing concentrations of X66 (left), or 20 μM X66 for indicated time (right). **F.** SK-BR-3 cells were pretreated with or without 10 μM MG132 for 1 h before exposure to 1 μM GM or 40 μM X66 for 12 h. Levels of HER2 and AKT were analyzed by Western blot.

The anti-proliferative activity of X66 *in vitro* was examined in several tumor cell lines. X66 inhibited the proliferation of SK-BR-3, BT-474, A549, K562 and HCT-116, in a concentration-dependent manner, with IC_50_ values of 8.9, 7.1, 7.5, 8.6 and 6.7 μM, respectively. (Figure 2A).

We further investigated the effect of X66 on cell cycle profile. X66, similar to GM, causes cell type-dependent cell cycle arrest. Treatment with 20 μM X66 resulted in G_1_ arrest in SK-BR-3 and A549 cells, with the proportion of cells in G_1_ phase increasing from 50.8% to 64.5% and 61.8% to 77.9%, respectively. However, 20 μM X66 arrested K562 cells in G_1_ plus G_2_/M phases, with the G_1_ fraction increasing from 34.0% to 45.1% and G_2_/M fraction increasing from 3.7% to 9.2%, respectively (Figure 2B). Furthermore, cell apoptosis was observed with prolonged X66 treatment for 48 h in SK-BR-3 cells. 20 μM X66 caused slightly cleavage of Poly (ADP-ribose) polymerase (PARP) Caspase-8, Caspase-9 and Caspase-3, and the phenomena became obvious at the concentration of 40 μM (Figure 2C). Together, these results indicate that X66 causes cell-cycle arrest followed by apoptosis.

### X66 decreases HSP90 client protein levels via the proteasome pathway

The HSP90 chaperone complex stabilizes many client proteins that play key roles in tumor formation and progression [24]. Therefore, we examined whether HSP90 inhibition by X66 can induce degradation of these oncoproteins. X66 effectively decreased the levels of specific oncogenic proteins, such as HER2, EGFR and BCR-ABL in BT-474, A549 and K562 cancer cell lines (Figure 2D). Similar effects were observed in SK-BR-3 cells. X66 treatment reduced the levels of HER2 and other client proteins including AKT, RAF-1, CDK6 and CDK4 in a concentration- and time-dependent manner (Figure 2E). The reduction of HSP90 client proteins was usually concurrent with induction of HSP72 and HSP27, a hallmark of HSP90 inhibition [25]. Unexpectedly, Both proteins were induced by GM, but not by X66, in all these tested cell lines (Figure 2D and 2E). X66 does not induce constitutive heat shock protein 70 (HSC70) either.

It has been proved that the HSP90 inhibitor-treated degradation of client proteins is mediated by proteasome [7, 8]. Co-treatment with proteasome inhibitor MG132 reversed the X66- and GM-induced degradation of HER2 and AKT (Figure 2F). The data suggest that X66 depletes HSP90 client proteins in a proteasome-dependent manner, and does not induce the expression of HSPs in various tumor cell lines.

### X66 does not induce HSR

**Figure 3:**
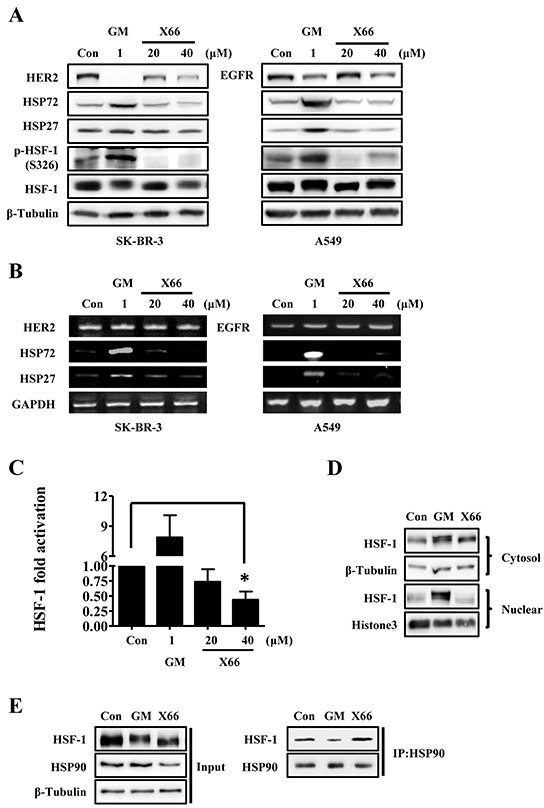
X66 does not induce HSR **A.** and **B.** SK-BR-3 and A549 cells were treated with indicated concentrations of GM or X66 for 8 h and 13 h, respectively. Cell lysates were analyzed by Western blot with indicated antibodies (A). Total mRNA was amplified by two-step RT-PCR and then separated on 1% agarose gel (B). **C.** SK-BR-3 cells were transiently transfected with a reporter plasmid encoding luciferase under the control of a HSE promoter or a plasmid encoding luciferase only. 48 h after transfection, SK-BR-3 cells were treated with indicated concentrations of GM or X66 for 8 h, and then assayed for luciferase activity. **D.** A549 cells were treated with GM or X66 for 13 h, and then were fractionated as described under “Materials and Methods”. The presence of HSF-1 was analyzed by Western blot. β-Tubulin and Histone3 were used as loading control of cytoplasmic fraction and nuclear fraction, respectively. **E.** A549 cells were treated with GM or X66 for 3 h, and then followed by co-immunoprecipation as described under “Materials and Methods”. Samples were analyzed by Western blot with indicated antibodies. *, *p* < 0.05.

We further examined the mechanistic action of X66 on HSP90 client protein and HSPs. First, we detected whether the changes of HSP90 client protein and HSPs observed after X66 treatment were reflected in the mRNAs. In SK-BR-3 cells, both X66 and GM declined the protein expression of HER2, and did not affect the mRNA expression. However, neither mRNA nor the protein of HSP72 and HSP27 was induced after X66 treatment, even at the concentration of 40 μM, whereas 1 μM GM triggered the transcription of these two HSPs prominently (Figure 3A and 3B). Similar results were obtained in A549 cells. These data indicate that X66 may not induce the transcription of stress-inducible heat shock elements (HSE)-containing genes.

HSF-1, the transcription factor for HSPs by recruiting to HSE, is critical for the induction of HSR [26]. We next investigated the effect of X66 on HSF-1 in SK-BR-3 cells that were transfected with a HSR reporter plasmid. The HSR reporter is a HSE-luciferase fusion construct, and the luciferase activity is coupled with the transcriptional activity of HSF-1. As shown in Figure 3C, X66 did not activate the activity of HSF-1, and partly inhibited the activity even at the concentration of 40 μM, whereas 1 μM GM triggered the activity significantly.

Previous studies have demonstrated that many steps are required for HSF-1 activation, including phosphorylation and accumulation in the nucleus [15, 27]. As expected, our results showed that a mobility shift for HSF-1, which is often correlated with the hyperphosphorylation, was observed after treatment with GM in SK-BR-3 and A549 cells (Figure 3A). In consistency with this, GM successfully phosphorylated HSF-1 at S326, which contributes significantly to the activation of HSF-1 [27]. On the contrary, X66 had little effect on the phosphorylation at S326 in either cell line, although it caused the mobility shift of HSF-1 at the concentration of 40 μM. We next investigated whether X66 has effect on the nuclear accumulation of HSF-1 in A549 cells. As indicated in Figure 3D, a marked accumulation of HSF-1 was found within the nuclear fraction in the cells treated with GM. In contrast, no increase in HSF-1 level within the nuclear fraction was observed after X66 treatment.

It is well-known that HSF-1 dissociates from HSP90 complex in cells after treatment with HSP90 inhibitors [12]. To find out whether X66 can cause the release of HSF-1 from HSP90 complex, we detected the protein level of HSF-1 by co-immunoprecipitation with HSP90 antibody. As shown in Figure 3E, HSF-1 that bound to HSP90 decreased in cells treated by GM but changed little after X66 treatment, compared with their respective control level. These data indicate that X66 is unable to release HSF-1 which binds to HSP90 complex and then results in its lack of HSR.

### X66 reverses the HSF-1 activator-induced HSR

Since X66 does not induce HSR, we were interested to find out whether its combination with HSF-1 activators, such as GM, MG132 or celastrol [28], has potential effect on anti-tumor activity. We evaluated the cellular activity of the combination treatment to the two agents alone by calculating the combination index (CI). The CI value definition for synergism is CI < 1, additive effect CI = 1, and antagonism CI > 1. As shown in Table 1, X66 showed synergistic effects in growth inhibition of all tested cell lines when combined with celastrol. Its combination with GM or MG132 also showed synergistic effects in some of the cell lines.

**Table 1:** Combination of X66 with HSP90 and proteasome inhibitors sensitizes tumor cells

Cell line	CI		
	GM	Celastrol	MG132
SK-BR-3	0.95 ± 0.04	0.89 ± 0.06	0.85 ± 0.12
A549	0.98 ± 0.04	0.87 ± 0.06	ND
HCT-116	0.96 ± 0.03	0.70 ± 0.06	ND
BT-474	ND	0.85 ± 0.05	0.96 ± 0.04

Cells were plated in 96-well plates and incubated with different concentrations of each compounds or their combinations for 72 h in triplicate. The concentration of X66 ranged from 0.625-10 μM. The fixed ratios of X66 to GM are 1000: 1(SK-BR-3), 500: 1(A549) and 250: 1(HCT-116), respectively. The fixed ratios of X66 to celastrol are 15: 1 (SK-BR-3, HCT-116), 5: 1 (A549) and 50: 1 (BT-474), respectively. The fixed ratios of X66 to MG132 are 25: 1 (SK-BR-3) and 7.5: 1(BT-474), respectively. The Calcusyn software was used to calculate the CI values. Values represent the mean ± SEM (n=3). ND, not determined.

**Figure 4:**
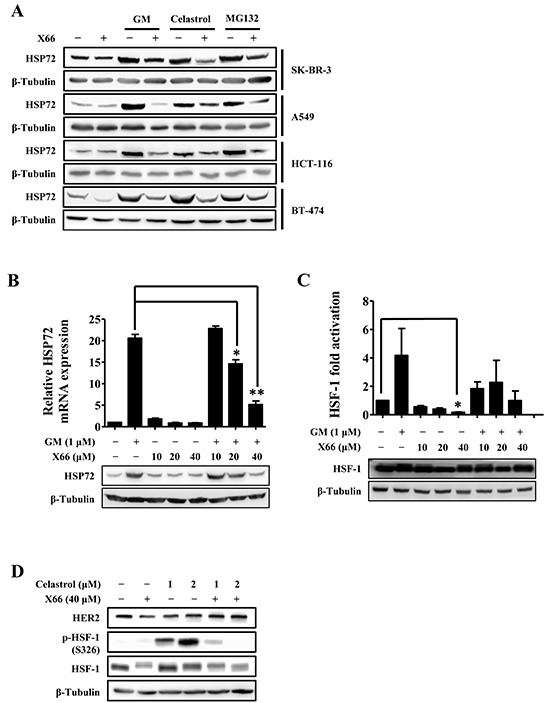
X66 blocks the HSF-1 activator-induced HSR **A.** SK-BR-3, A549, HCT-116 and BT-474 cells were incubated with or without X66 for 1 h before exposed to GM, celastrol or MG132 for 8 h. Cell lysates were analyzed by Western blot with indicated antibodies. **B.** SK-BR-3 cells were pretreated with or without increasing concentrations of X66 for 1 h before exposed to GM for 8 h. The protein and mRNA levels of HSP72 were analyzed by Western blot and Real-time PCR, respectively. **C.** SK-BR-3 cells were transiently transfected with a reporter plasmid encoding luciferase under the control of a HSE promoter or a plasmid encoding luciferase only. 48 h after transfection, the cells were treated similarly as described in B and assayed for luciferase activity. The protein level of HSF-1 were analyzed by Western blot **D.** BT-474 cells were pretreated with or without X66 for 1 h before exposed to celastrol for 8 h. Cell lysates were analyzed by Western blot with indicated antibodies. *, *p* < 0.05; **, *p* < 0.01.

To examine the mechanistic basis for this synergistic interaction, we investigated the effect of X66 on the stress response, which is an important determinant of sensitivity to these tested agents. As shown in Figure 4A, X66 blocked the up-regulation of HSP72 protein induced by GM, celastrol or MG132 in all the tested cell lines including SK-BR-3, A549, HCT-116 and BT-474 cells. In consistency with this data, X66 abrogated the GM-induced transcription of HSP72 in a concentration-dependent manner (Figure 4B). The capability of reversing the GM-induced HSR was further confirmed by monitoring the transcriptional activity of HSF-1, while the protein levels of HSF-1 changed little after combination treatment (Figure 4C). Moreover, the hyperphosphorylation of HSF-1 at S326 induced by celastrol was inhibited by X66 markedly, while the protein level of HER2 was restored (Figure 4D). Thus, these results suggest that the enhanced anti-proliferation activity of X66 in combination with these agents *in vitro* might be attributed to its capability of reversing HSR through diminishing the transcriptional activity of HSF-1.

### The *in vivo* study of X66

#### Pharmacokinetic/pharmacodynamic relationship of X66 in tumor-bearing animals

**Figure 5:**
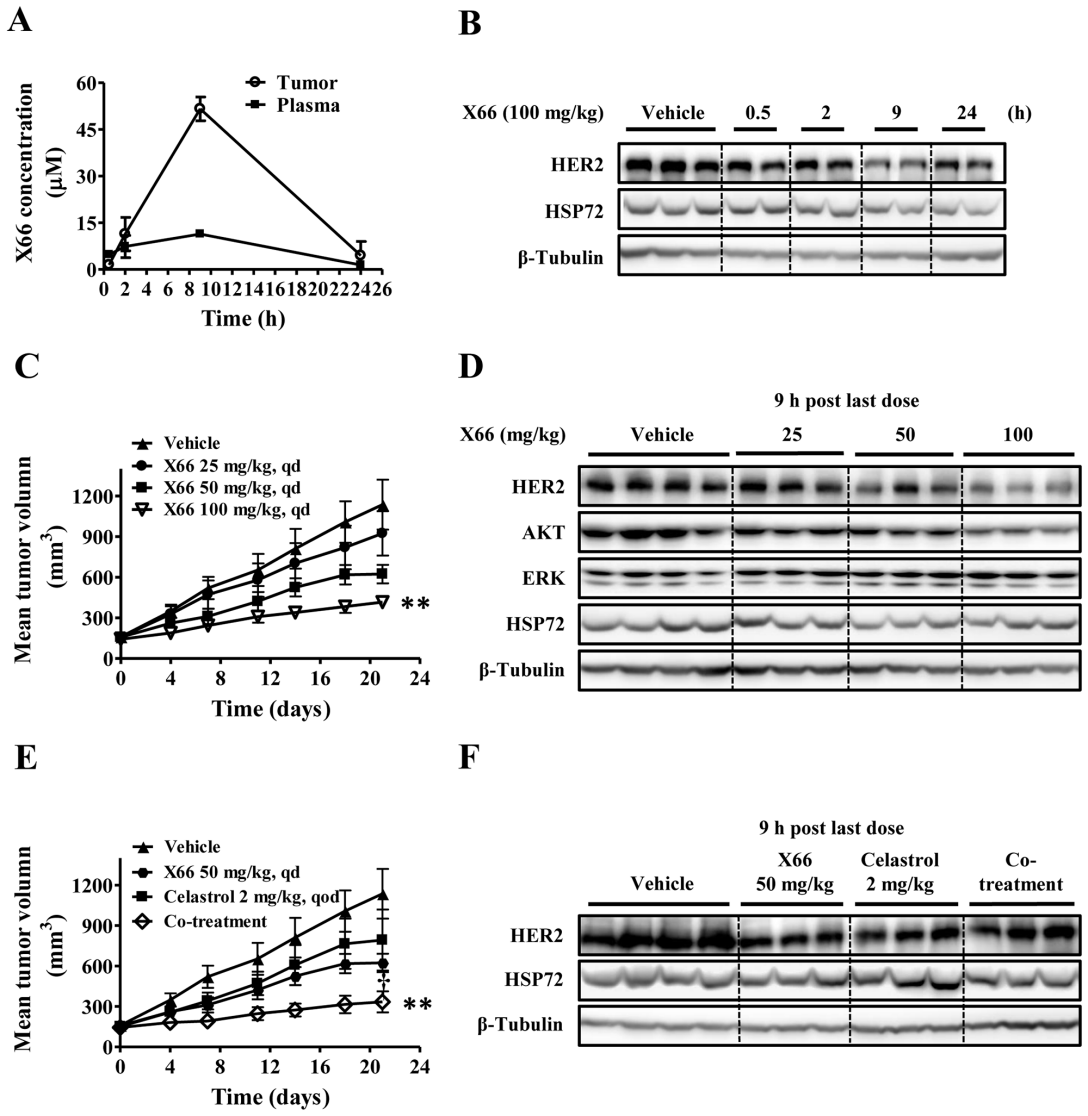
*In vivo* study of X66 **A.** and **B.** BT-474 tumor-bearing mice were received a single dose of 100 mg/kg X66 i.p., and the mice were sacrificed at indicated time. The concentrations of X66 were determined in blood plasma and tumor tissues (A). In parallel, tumor extracts were used to analyze HER2 and HSP72 levels by Western blot (B). **C.** and **D.** BT-474 tumor-bearing mice were received vehicle (n=10) or X66 (n=6) i.p. daily for 21 days at indicated doses. Tumor volumes were measured (C). The mice were sacrificed 9 h after last dose, and tumor was removed and analyzed by Western blot (D). **E.** and **F.** BT-474 tumor-bearing mice were received vehicle (n=10), X66 50 mg/kg, celastrol 2 mg/kg, or their combination (n=6). Tumor volumes were measured (E). The mice were sacrificed 9 h after last dose, and tumor was removed and analyzed by Western blot (F). Data are presented as mean ± SEM. **, *p* < 0.01 verses vehicle; †, *p* < 0.05 verses either single agent alone.

Given its encouraging activity *in vitro*, we investigated the antitumor efficacy of X66 *in vivo*. A single-dose pharmacokinetic/pharmacodynamic study was first conducted in the HER2-overexpressing BT-474 breast cancer xenograft model. After a single intraperitoneal injection (i.p.) dosing of X66 at 100 mg/kg, the plasma and tumor were collected at various time points over a 24-h period. A portion of the tumor tissue was prepared for pharmacokinetic evaluation, and the remaining tumor tissue was subjected to Western blot analysis. As shown in Figure 5B, HER2 was inhibited from 0.5 to 24 h after compound administration in a time-dependent manner, which was in concordance with the changes of the plasma and the intratumoral concentrations of X66 (Figure 5A). The protein level of HER2 decreased significantly at 9 h, correlating with the plasma and tumor exposure to X66 with the concentration of 11.4 μM and 51.6 μM, respectively. Note that the protein level of HSP72 in tumor tissue remained stable during the time course, which was consistent with *in vitro* results.

#### X66 exhibits potent antitumor activity accompanied by degradation of HSP90 client proteins *in vivo*

We next evaluated the antitumor efficacy of the compound given i.p. daily against the same BT-474 xenograft model. X66 exhibited dose-dependent antitumor activity with the treated/control (T/C) value of 78.4%, 47.7% and 28.0% for doses of 25, 50 and 100 mg/kg, respectively, on the final treatment day (Figure 5C). These treatments were well tolerated, and no significant body weight loss was observed during the course of the experiment in all groups (data not shown). Furthermore, the antitumor activity in this model was correlated with the reduced levels of HSP90 client proteins, including HER2 and AKT in the tumor tissues (Figure 5D). Neither HSP72 nor non-client protein ERK had changed.

#### X66 enhanced anti-tumor activity of celastrol in xenograft model

To determine whether the synergistic anti-proliferative activity of X66 in combination with celastrol translated *in vivo*, we assessed the combinational treatment against BT-474 xenograft model. As shown in Figure 5E, treatment with 2 mg/kg celastrol alone resulted in moderate block of tumor growth (T/C of 65.6%), whereas the addition of 50 mg/kg X66 was significantly more potent than each single agent in inhibiting the growth of tumor xenografts with a T/C value of 19.5% (*p* < 0.01). No additional toxicity was observed in the co-treatment group as assessed by treatment-related mortality and body weight change (data not shown).

It was noted that the induction of HSP72 in the co-treatment group became less prominent compared with the celastrol treated group in tumor tissues, while HER2 did not decrease in the co-treatment group compared with the monotherapy groups (Figure 5F). Altogether, these preliminary *in vivo* results suggest that X66 causes disease stasis as a single agent and can enhance the efficacy of other anticancer agents when used in combination studies.

## DISCUSSION

In this study, we demonstrated that X66 was a potent inhibitor of HSP90 that structurally differed from all the well-known HSP90 inhibitors, such as GM and NVP-AUY922. The results of SPR and pull-down assay showed that X66 bound to human HSP90, and the HSP90 binding assay showed its bind site located in N-terminal domain. However, in two different competition assays, the binding of X66 to the N-terminal domain of HSP90 could not be competed by GM or NVP-AUY922 and *vice versa*. Molecular modeling of the HSP90-X66 complex indicates that X66 adopts a very different binding mode from GM (Supplementary Figure S1). GM almost fills the whole ATP pocket [6], while X66 just occupies partial the pocket and extends into the adjacent hydrophobic subpocket with pyrazole moiety. Previous study has proved that a triazine derivative CH5138303 almost occupies the whole ATP pocket as GM does [29]. Thus, the data indicate that X66 possesses a unique binding site of HSP90 which is distinct from that of classic HSP90 inhibitors.

Previous studies have indicated that the up-regulation of stress-inducible proteins, particularly HSP72 and HSP27, after HSP90 inhibition, might be responsible for the poor activity observed in HSP90 inhibitors clinical trials [24, 30, 31]. Furthermore, HSF-1, the master regulator of HSR, is believed to correlate with tumorigenesis and be the potential target of cancer therapy [32, 33]. Therefore, targeting HSP90 without induction of HSR represents a new direction for HSP90 inhibitors development. Several compounds have been reported inhibit HSP90 function in the absence of HSR [34–36]. The SMX inhibitors derived from sansalvamide A modulate the C-terminus via binding to the N and middle domain [37–39]. The compounds do not induce HSR, and decrease the protein level of HSF-1, and the mRNA and protein levels of HSPs [40, 41].

It is well-established that when GM binds to HSP90, the suppression of HSF-1 by HSP90 complex is disrupted [12]. Released from the HSP90 complex, the monomeric HSF-1 goes through trimerization, nuclear accumulation and post-modification, and then become fully activated [14, 15]. Our data showed that X66 had no effects on interfering the interaction between HSP90 and HSF-1, causing accumulation of HSF-1 in nuclear fraction, increasing the stress-inducible phosphorylation at S326, or triggering its transcriptional activity. Thus, we presume that the unique binding mode between X66 and HSP90, which has little impact on the binding between HSP90 and HSF-1, may be responsible for its lack of HSR. Our results also show that X66 can reverse the HSR induced by HSF-1 activators, of which the probable mechanism is that it inhibits HSF-1 transcriptional activity by repressing the phosphorylation at S326.

Several studies have proved that targeting HSR by inhibition of HSP72, HSP27 or HSF-1 can sensitize cancer cells to HSP90 and proteasome inhibitors [18, 42, 43]. Our data show that the combination treatments of X66 with GM, celastrol or MG132 resulted in enhanced anti-proliferative effects in the tested cancer cell lines. Celastrol, which targets both HSP90 complex and proteasome and leads to the degradation of HSP90 client proteins and induction of HSR, acts synergistically with GM and 17-AAG in cell-growth inhibition [44, 45]. We observed that the up-regulation of HSP72 triggered by celastrol was counteracted by X66, whereas the protein level of HER2 changed little in the combinational group compared to the control group both *in vitro* and *in vivo*. These results indicate that the ability of X66 to blockade HSR may attribute to the enhanced anti-tumor efficacy of the combinational treatments.

In conclusion, X66, a novel HSP90 inhibitor, which binds to the N-terminal domain of HSP90, causes the degradation of client proteins and growth inhibition of tumor cells both *in vitro* and *in vivo*. In particular, X66 does not induce HSR, instead, it reverses the HSF-1 activator-trigged HSR, which provides the possibility for combination treatment of X66 and overcoming HSP90 inhibitor-resistance in clinical trials. The discovery of X66 is promising as it points to the exploration of a whole new series of HSP90 inhibitors, and provides a new strategy of combination treatment in cancer therapy.

## MATERIALS AND METHODS

### Chemical information

Synthesis and characterization of X66 and Biotin-X66 are provided in the Supplementary Methods.

### Reagents and antibodies

GM was obtained from Sangon Biotech (Shanghai, China). Celastrol was purchased from Selleck Chemicals (Houston, TX, USA). NVP-AUY922 was purchased from Meilun Biology Technology (Dalian, China). MG132 and antibody to β-tubulin were from Sigma-Aldrich (St. Louis, MO, USA). FITC-GM, human HSP90α and antibodies specific for HSF-1 and HSP72 were from Enzo life sciences (Farmingdale, NY, USA). Antibodies to p-HSF-1 (S326), HSC70 and HSP90 were from Abcam (Cambridge, UK). Antibodies specific for His-tag, HER2, EGFR, AKT, CDK4 CDK6, PARP Procaspase-3, cleaved caspase3, Caspase-8 and Caspase-9 were purchased from Cell Signalling Technology (Beverly, MA, USA). Antibodies to RAF-1 and ERK were from Santa Cruz Biotechnology (Santa Cruz, CA, USA).

### Cell culture

The cancer cell lines SK-BR-3, BT-474, A549, K562 and HCT-116 were purchased from the American Type Culture Collection (Manassas, VA, USA). Cells were cultured according to instructions provided by the American Type Culture Collection. All the cells have been tested and authenticated by Genesky Biotechnologies. Inc. (Shanghai, China) using fluorescent amplified restriction fragment polymorphism (FAFLP) method.

### SPR assay

Measurements of SPR were performed as described previously [46]. Briefly, using a BIA-core 3000 (GE healthcare, Cleveland, OH, USA) instrument, HSP90α (150 μg/ml in 10 mM sodium acetate pH 4.3) was immobilized on the CM5 sensor chip surface using standard amine-coupling protocols to obtain densities of 16 kRU. Unreactive groups were then quenched with 1 M ethanolamine. Compounds were diluted in HBS-EP (20 mM HEPES, pH 7.4, 150 mM NaCl, 0.3 mM EDTA, 0.05% P20), and injected from lowest to highest concentrations at a flow rate of 30 μl/min for 1 min. And the dissociation of compound-protein complex was monitored for an additional 3 min.

### FP assay

Competitive binding of inhibitors to HSP90α was monitored using FITC-GM in a FP assay [47]. Briefly, compound dilutions were incubated with HSP90α (90 nM final) and FITC-GM (2 nM final) in a 96-well microplate at 4°C for 16 h in the presence of assay buffer (20 mM HEPES, pH 7.4, 50 mM KCl, 5 mM MgCl_2_, 20 mM NaMoO_4_, 0.01% NP40, 2 mM DTT and 0.1 mg/ml BSA). Polarization was measured in Synergy H4 Hybrid reader (BioTek, Winooski, VT, USA) using Gen5.0 software (BioTek). [49]

### Western blot

Cells pellets were prepared in lysis buffer (100 mM Tris–HCl, pH 6.8, 2% SDS, 20% glycerol, and 1 mM DTT). Equal amounts of whole cell lysates were separated by SDS-PAGE, and electroblotted onto Immobilon PVDF membranes (Millipore, Bedford, MA, USA). Proteins were detected by immunoblotting using the Western blot image System (Clinx Science Instruments, Shanghai, China) with the ChemiCapture software.

### Pull-down assay and HSP90 binding assay

Purified full-length, N-terminal fragment, C-terminal fragment of HSP90α or cell lysates in lysis buffer (20 mM HEPES, pH 7.3, 1 mM EDTA, 5 mM MgCl_2_, 100 mM KCl) were incubated with Biotin-X66 for 3 h at 4°C, and then incubated with Streptavidin Plus UltraLink Resin (Thermo scientific, Pittsburgh, PA, USA) overnight at 4°C. The beads were washed three times in binding buffer and heated for 10 min at 95°C in SDS-PAGE sample buffer. Samples were analyzed by Coomassie blue staining or Western blot using His-tag or HSP90 antibody.

### Cytotoxicity assay

Cell growth inhibition was determined using a sulforhodamine B (SRB) assay or 3-(4,5-dimethylthiazol-2-yl)-2,5-diphenyl tetrasodium bromide (MTT) assay as described previously [48, 49]. Curve-fitting software GraphPad Prism version 5 (GraphPad Software, San Diego, CA, USA) was used to calculate IC_50_ values.

### RT-PCR and qPCR

Total RNA was harvested from cells using Trizol reagent (TAKARA, Dalian, China) and then reverse transcribed to cDNA using the PrimeScript^TM^ Reverse Transcription reagent kit (TAKARA) according to the manufacturer's instructions. PCR was performed using premix Taq (TAKARA) on a T100 Thermal Cycler PCR System (Bio-Rad Laboratories, Hercules, CA, USA). qPCR was performed using a SYBR Premix Ex Taq (TAKARA) on the StepOnePlus Real-Time PCR System (Applied Biosystems, FosterCity, CA, USA). The PCRs were performed using primers as follows: HSP72, 5′-AGAGCCGAGCCGACAGAG-3′ (forward) and 5′-CACCTTGCCGTGTTGGAA-3′ (reverse); HSP27, 5′-GGACGAGCATGGCTACATCT-3′ (forward) and 5′-GACTGGGATGGTGATCTCGT-3′ (reverse); HER2, 5′-CTGCACCCACTCCTGTGTGGACCTG-3′(forward) and 5′-CTGCCGTCGCTTGATGAGGATC-3′ (reverse); EGFR, 5′-GCCAAGGCACGAGTAACAAGC-3′ (forward) and 5′-AGGGCAATGAGGACATAACC-3′ (reverse); GAPDH, 5′-GGGGAAGGTGAAGGTCGGAGTC-3′ (forward) and 5′-CAAGCTTCCCGTTCTCAGCCTT-3′ (reverse). In qPCR, GAPDH was used as a normalized control.

### Transfection and luciferase assay

The Cignal Heat Shock Response Reporter (luc) Kit was purchased from QIAGEN (Hilden, German). Transfection was performed with cells exponential growth using Lipofectamine 2000 (Life technologies, Carlsbad, CA, USA). 48 h after transfection, the cells were treated with different concentrations of inhibitors for 8 h. The cells were lysed and luciferase activity was measured using Synergy H4 Hybrid reader (BioTek) as described previously [50].

### Subcellular protein fractionation

This procedure is a modification of the one described previously [51]. Briefly, cells were pelleted, and lysed in NP40 low salt buffer (10 mM HEPES, pH 7.9, 1 mM EDTA, 1% NP40, 10 mM KCl, 0.1 mM DTT and 1% cocktail protease inhibitor) on ice. The lysate was vortexed and sedimented at 14000 × g for 1 min. The supernatant was collected as cytoplasmic fraction. Nuclei were re-suspended in high salt buffer (20 mM HEPES, pH 7.9, 1 mM EDTA, 25% glycerol, 400 mM NaCl, 0.1 mM DTT and 1% cocktail protease inhibitor), vortexed for 30 min at 4°C, and sedimented at 14000 × g for 5 min. The supernatant was the nuclear fraction.

### Co-immunoprecipitation

After treated with dithiobs (succinimidyl propionate) as described previously [12], cell lysates were incubated with antibody for 3 h at 4°C. Incubation was continued overnight after addition of 30 μl protein-G Sepharose beads (50% slurry) (Thermo scientific) at 4°C. The beads were washed three times in binding buffer and heated for 10 min at 95°C in SDS-PAGE sample buffer. Samples were analyzed by Western blot.

### *In vivo* study

Female nude mice (Balb/cA-nude, 5-6 week old) were purchased from Shanghai SLAC Laboratory Animal Co., Ltd (Shanghai, China). Human tumor xenografts of BT-474 cells were implanted subcutaneously in the right flank of animal. When tumor volumes reached 100-200 mm^3^, the mice were randomized to receive vehicle control (10 mice/group) or tested compounds (6 mice/group). X66 was administrated i.p. daily at the dose of 25, 50 or 100 mg/kg for 21 days, celastrol was administrated i.p. every other day for 10 times at the dose of 2 mg/kg and the co-treatment group was administrated with celastrol (2 mg/kg) every other day and X66 (50 mg/kg) daily. Tumor volume was calculated as (length × width^2^)/2. The therapeutic effect of a given compound was expressed in terms of T/C %, which was calculated as: T/C (%) = (V_t_′− V_t_)/(V_c_′− V_c_) ×100%, where V_t_′ and V_t_ are the volumes of treated group on each day of measurement and on the day of initial treatment, respectively. And V_c_′ and V_c_ are the volumes of vehicle group on each day of measurement and on the day of initial treatment, respectively.

Pharmacokinetic/pharmacodynamic studies were carried out as described previously [49]. Mice bearing BT-474 tumors received a single i.p. of 100 mg/kg X66 or vehicle, and then tumor tissue and blood were collected at different times post-dosing. Concentrations of X66 in plasma and tissue were determined by HPLC/tandem mass spectrometry. Tumor samples were subjected to Western blot analysis.

Animal experiments were carried out in accordance with the Institutional Animal Care and Use Committee guidelines at the Shanghai Institute of Materia Medica, Chinese Academy of Sciences.

### Data analysis

Data were presented as the means ± SEM and were plotted using GraphPad Prism Version 5. A paired two-tailed Student's t-test was used to test for significance where indicated. Differences were considered significant at *p* < 0.05.

## SUPPLEMENTARY FIGURES AND TABLES


