# Supplementary material for: X66, a novel N-terminal heat shock protein 90 inhibitor, exerts antitumor effects without induction of heat shock response
Source: Oncotarget. 2016 Apr 18;7(20):29648–63. doi: 10.18632/oncotarget.8818 (PMC5045423; doi:10.18632/oncotarget.8818)
Supplement: Supplementary file 1 [file oncotarget-07-29648-s001.pdf]

SUPPLEMENTARY METHODS

Synthesis and characterization of X66 and Biotin-X66

General: All starting materials and solvents were purchased from commercial suppliers and used without further purification. Reactions were monitored by thin layer chromatography (TLC) of silica gel (HSGF254) at 254 nm wavelength. The LRMS and HRMS were recorded on Finnigan LCQ/DECA and Micromass Ultra Q-TOF (ESI) spectrometer, respectively.

The ^{1}H and ^{13}C spectra were taken on Bruker Avance - 400 and 500 NMR spectrometer operating at 400 MHz for ^{1}H and 126 MHz for^{13}CNMR, respectively, using TMS as the internal standard and $CDCl_3$ or DMSO-d_6 as the solvent. Chemical shifts are given in δ values of ppm. The abbreviations s is singlet, d is doublet, t is triplet and m is multiplet. Coupling constants (*J*) were measured in hertz (Hz).

^{1}H and ^{13}C spectrums of X-66 and Biotin-X66 exhibited special signals while using $CDCl_3$ or DMSO-d_6 as solvent, which may be due to the structural resonance. The special signals are indicated by *major* or *minor*.

2,4,6-trichloro-1,3,5-triazine was coupled with 4-bromoaniline using $NaHCO_3$ to give intermediate **2** which reacted with 3,5-dimethyl-1H-pyrazole to provide the compound **3**. The chloride of **3** was substituted by hydrazine hydrate. Intermediate **4** had a dehydration with indole-3-aldehyde and biotin substituted indole-3-aldehyde **6** to give the desired imine X66 and Biotin-X66**,** respectively.

4-(2-((1H-indol-3-yl)methylene)hydrazinyl)-N-(4-bromophenyl)-6-(3,5-dimethyl-1H-pyrazol-1-yl)-1,3,5-triazin-2-amine (X66)

^{1}H NMR (400 MHz, DMSO-d_6) δ 11.60 (s, 1H, major), 11.55 (s, 1H, minor), 11.50 (s, 1H, major), 11.27 (s, 1H, minor), 10.15 (s, 1H), 8.54 – 8.34 (m, 2H), 8.27 – 7.74 (m, 3H), 7.64 – 7.37 (m, 3H), 7.39 – 7.03 (m, 2H), 6.20 (s, 1H, minor), 6.15 (s, 1H, major), 2.92 (s, 3H, minor), 2.69 (s, 3H, major), 2.22 (s, 3H, minor), 2.20 (s, 3H, major).

^{13}C NMR (126 MHz, DMSO) δ 165.14 (major), 164.79 (minor), 164.39, 163.77 (minor), 163.15 (major), 150.27 (minor), 150.05 (major), 143.59 (minor), 143.19 (major), 142.83, 139.88 (minor), 139.72 (major), 137.66 (major), 137.46 (minor), 131.67 (minor), 131.57 (major), 130.90 (major), 130.52 (minor), 124.71, 123.17 (major), 123.06 (minor), 122.57 (minor), 122.44 (major), 122.23, 120.54, 114.32 (major), 114.23 (minor), 112.42 (major), 112.26 (minor), 110.62 (minor), 110.42 (major), 15.94 (minor), 15.70 (major), 13.97 (minor), 13.90 (major).

HRMS (ESI): calcd. for $C_{23}H_{21}N_9Br$ $[M+H]^+$: 502.1103, found: 502.1100.

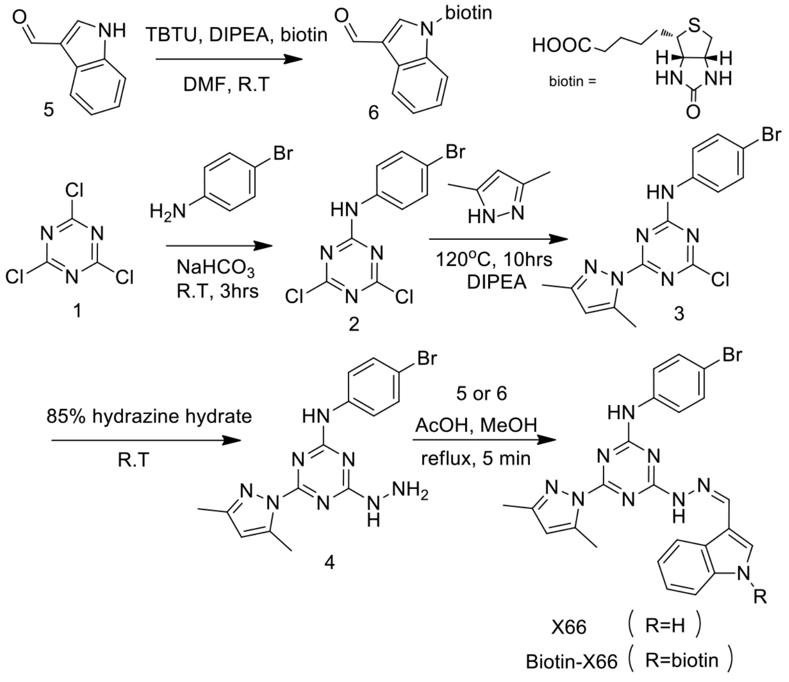

Scheme 1: Synthetic route for preparing X66 and X66-biotin.

(3aS,4S,6aR)-4-(5-(3-((2-(4-((4-bromophenyl)amino)-6-(3,5-dimethyl-1H-pyrazol-1-yl)-1,3,5-triazin-2-yl)hydrazono)methyl)-1H-indol-1-yl)-5-oxopentyl)tetrahydro-1H-thieno[3,4-d]imidazol-2(3H)-one (Biotin-X66)

^{1}H NMR (400 MHz, DMSO-d_6) δ 11.84 (s, 1H, major), 11.62 (s, 1H, minor), 10.25 (d, J = 32.1 Hz, 1H), 8.79 – 8.31 (m, 4H), 8.19 – 7.75 (m, 2H), 7.64 – 7.18 (m, 4H), 6.50 (s, 1H), 6.40 (s, 1H), 6.21 (s, 1H, minor), 6.16 (s, 1H, major), 4.32 (dd, J = 7.3, 4.8 Hz, 1H), 4.17 (dd, J = 7.3, 4.4 Hz, 1H), 3.21 – 3.02 (m, 3H), 2.91 (s, 3H, minor), 2.84 (dd, J = 12.4, 5.1 Hz, 1H), 2.70 (s, 3H, major), 2.61 (s, 1H, major), 2.58 (s, 1H, minor), 2.22 (s, 3H, minor), 2.20 (s, 3H, major), 1.83 – 1.65 (m, 3H), 1.62 – 1.40 (m, 3H).

^{13}C NMR (126 MHz, DMSO-d_6) δ 172.59, 165.17, 164.87 (minor), 164.76 (major), 163.80, 163.25 (minor), 163.19 (major), 150.46 (minor), 150.27 (major), 143.58 (minor), 143.34 (major), 140.60 (major), 140.43 (minor), 139.54, 136.54 (major), 136.35 (minor), 131.72 (minor), 131.62 (major), 130.52 (major), 130.12 (minor), 127.20, 126.26, 124.18, 123.22 (minor), 122.94 (major), 122.68 (major), 122.37 (minor), 117.68, 116.53 (major), 116.45 (minor), 114.56 (major), 114.49 (minor), 110.75 (minor), 110.58 (major), 61.50, 59.65, 55.91, 40.39, 35.19, 28.52, 28.49, 24.52, 15.91 (minor), 15.77 (major), 13.91.

HRMS (ESI): calcd. for $C_{33}H_{34}N_{11}O_2NaSBr$ $[M+Na]^+$: 750.1699, found: 750.1720.

Plasmids construction

The His-HSP90α expression vector (pET28a-HSP90α) was constructed by first amplifying the full-length human HSP90 cDNA from pUC-HSP90α by PCR, and subcloned into pET28a using *Sal*I and *Not*I. Subsequently, the regions coding of amino acids 1-236 and 629-732 which correspond to the N-terminal domain and C-terminal domain of the full-length protein, respectively, was amplified by PCR, and subcloned back into pET28a using *EcoR*I and *Xho*I to produce pET28a-HSP90NT and pET28a-HSP90CT.

Protein purification

The BL21-CondonPlus (DE3)-RIL-X strain was used as the host strain for expression of the full-length human HSP90α, fragment of N-terminal domain and fragment of C-terminal domain. An overnight culture of E.coli carrying HSP90α, N-terminal fragment, C-terminal fragment or yeast HSP90 was used to inoculate LB containing 50 μg/ml kanamycin or 100 μg/ml ampicillin. The culture was grown to an OD_{600} of 0.8, induced with 1mM IPTG and grown for 3 h before collecting by centrifugation. Cells were re-suspended in buffer A (20 mM HEPES-HCl pH 8.0, 0.5 M NaCl), 8 mM imidazole and 15% glycerol. Cells were disrupted by sonication in the presence of protease inhibitors (Roche life science, Indianapolis, IN, USA) and the lysate spun at 18000 × g at 4°C for 60 min. The supernatant was loaded onto a NI-NTA column (QIAGEN) which was equilibrated with buffer A containing 8 mM imidazole. The bound His-tagged protein was subsequently eluted with buffer A containing 100-300 mM imidazole, and concentrated using Amicon Ultra-15 centrifugal filter (Millipore). The concentrated protein was diluted with buffer B (20 mM HEPES-HCl pH 7.4) and loaded onto Q-sepharose column (GE healthcare). A linear gradient of NaCl (0-1 M) in buffer B was applied to the column for protein elution. The fraction was dialyzed in 20 mM HEPES-HCl pH 7.4 containing 150 mM NaCl and concentrated using Amicon Ultra-15 centrifugal filter. All proteins were store at -80°C.

Cell cycle analysis

Cells were collected, washed with PBS twice, and fixed with 70% ethanol overnight at -20°C. After RNaseA incubation and propidium iodide staining, cells were analyzed using a FACScan flow cytometer (BD Biosciences, San Jose, CA, US) with CellQuest and ModiFit LT3.0 software.

LC-MS/MS-based bioanalytical assays

Validated bioanalytical methods were used to measure test compounds in plasma and tumor tissue using a API4000 instrument (Applied Biosystems, Foster, CA, USA) interfaced via an electrospray ionization probe with a liquid chromatography (Agilent Technologies, Waldbronn, Germany). Chromatographic separations for measurement of X66 were achieved on a Phenomenex Gemini 5-μm C_{18} column (50 × 2.0 mm, Torrance, CA, USA). Mobile phases for measurement of X66 were acetonitrile (containing 0.1% formic acid; solvent A) and water (containing 0.1% formic acid and 5 mM ammonium acetate; solvent B). A binary escalation gradient elution was performed, which consisted of three isocratic segments, i.e., 0 to 0.5 min at 70% B, 0.9 to 1.9 min at 10% B, and 2 to 3.5 min at 70% B. MS/MS was performed in the positive ion mode using the precursor-to-product ion pairs m/z 502.8→407.1 and 502.8→386.4 for X66 in plasma and tumor tissue, respectively, and m/z 324 →127 for the internal standard (IS) gliclazide in both plasma and tumor tissue.

The LC effluent flow was introduced into the electrospray ionization probe at a flow rate of 0.5 $ml \cdot min^{-1}$. To a 25 μl aliquot of plasma sample, 25 μl of

internal standard (50.0 ng/ml, gliclazide), and 100 µl of acetonitrile were added. To a 80 µl aliquot of tumor tissue sample, 20 µl of IS, and 100 µl of acetonitrile were added. After votex-mixing for 1 min and centrifugation at 14000 rpm for 5 min, the supernatant (20 µl for plasma and 10µl for tumor tissue, respectively) was applied to LC-MS/MS-based analysis. Matrix-matched calibration curves were constructed for the X66 using weighted (1/X) linear regressions of the compound response (peak area; Y) against the corresponding nominal concentrations.

Molecular docking

The X-ray crystal structure of the N-terminus domain of HSP90 (PDB code: 2YK9) [1] was retrieved from the Protein Data Bank. Molecular docking was performed in standard procedure by Glide v5.6 in its SP mode [2, 3]. LigPrep v2.4 was utilized to pre-process the compound using default parameters.

REFERENCES

1. Vallee F, Carrez C, Pilorge F, Dupuy A, Parent A, Bertin L, Thompson F, Ferrari P, Fassy F, Lamberton A, Thomas A, Arrebola R, Guerif S, Rohaut A, Certal V, Ruxer JM, et al. Tricyclic series of heat shock protein 90 (Hsp90) inhibitors part I: discovery of tricyclic imidazo[4,5-c]pyridines as potent inhibitors of the Hsp90 molecular chaperone. Journal of medicinal chemistry. 2011; 54:7206-7219.
2. Friesner RA, Banks JL, Murphy RB, Halgren TA, Klicic JJ, Mainz DT, Repasky MP, Knoll EH, Shelley M, Perry JK, Shaw DE, Francis P and Shenkin PS. Glide: a new approach for rapid, accurate docking and scoring. 1. Method and assessment of docking accuracy. Journal of medicinal chemistry. 2004; 47:1739-1749.
3. Halgren TA, Murphy RB, Friesner RA, Beard HS, Frye LL, Pollard WT and Banks JL. Glide: a new approach for rapid, accurate docking and scoring. 2. Enrichment factors in database screening. Journal of medicinal chemistry. 2004; 47:1750-1759.

SUPPLEMENTARY FIGURE AND TABLES

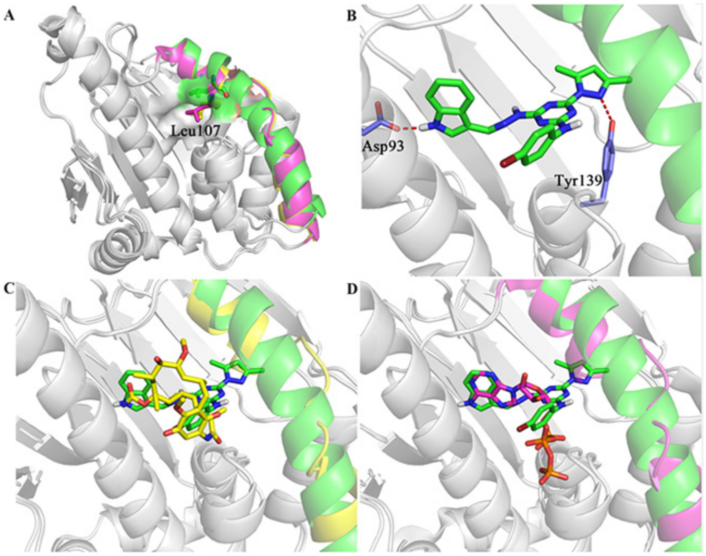

Supplementary Figure S1: Predicted binding mode of X66 to HSP90. A. The superimposition of three crystal structures of the N-terminal domain of HSP90 (PDB ID: 2YK9, 1YET and 3T0Z). **B.** X66 binds to the N-terminal domain of HSP90 (PDB ID: 2YK9) in the docking study. **C.** The docked complex structure of X66 is superimposed on the crystal structure of GM binding to HSP90 (PDB ID: 1YET). **D.** The docked complex structure of X66 is superimposed on the crystal structure of ATP binding to HSP90 (PDB ID: 3T0Z). The cartoon form of HSP90 is shown. The helix beside/occupying the hydrophobic subpocket and the stick form of Leu107 in 2YK9, 1YET and 3T0Z are colored in green, yellow and magenta, respectively, with the rest parts in gray. The hydrophobic subpocket in 2YK9 are displayed by protein surface. The dashed lines in red represent the interactions between X66 and HSP90. The stick form of Asp93 and Tyr139 is displayed with carbon atoms colored in purple. X66, GM and ATP are shown in sticks with the carbon atoms colored in green, yellow and magenta, respectively.

Supplementary Table S1: Quality control of plasma

Nominal concentration (ng/ml)	Measured concentration (ng/ml)	Accuracy RE (%)	Precision RSD (%)
30	28.9 ± 5.9	-3.67	20.4
300	296± 2.1	-1.33	0.70
3000	2960± 240	-1.33	8.11
30000	28950± 2899	-3.50	10.0

Supplementary Table S2: Quality control of tumor tissue

Nominal concentration (ng/g)	Measured concentration (ng/g)	Accuracy RE (%)	Precision RSD (%)
200	197 ± 23.3	-1.50	11.8
2000	1940 ± 297	-3.00	15.3
5000	4350 ± 325	-13.0	7.47
24000	22200 ± 1556	-7.50	7.07
